# Vernacular english literacy practices among language students using AI: A case study in Saudi Arabia

**DOI:** 10.1371/journal.pone.0358237

**Published:** 2026-09-15

**Authors:** Nada Bin Dahmash

**Affiliations:** Department of Humanities, College of Applied Studies, King Saud University, Riyadh, Kingdom of Saudi Arabia; Kalasalingam Academy of Research and Education, INDIA

## Abstract

Recent developments in Artificial Intelligence (AI) have encouraged teachers of English as a Foreign Language (EFL) to evaluate its potential benefits to the process of language learning. However, little is currently known about how language learners use AI outside the EFL classroom. Accordingly, this study addresses the underexplored area of informal, AI-mediated English literacy practices among EFL learners. Data from a qualitative case study involving 12 postgraduate students who participated in a focus group and retrospective interviews at a Saudi Arabian university were analyzed. Content analysis revealed the students engaged in English literacy practices using AI, and that these were categorized according to their usage in three domains: (1) practices for English language development, (2) to assist with daily life, and (3) to benefit academic studies. Approximately 50% of AI engagement in the English language aimed to practice the participants’ English reading, writing, and speaking skills, while leveraging customized learning opportunities. In addition, examples serving daily life functions were related to personal and vocational activities, and those serving academic life focused on preparing for examinations, academic research, and university assignments. The study therefore defined the concept of AI-mediated English literacy practices, ultimately providing insights into how language students engage in informal AI-mediated literacy practices in EFL settings. Thus, the findings reveal valuable pedagogical insights to inform EFL teachers.

## Introduction

Artificial Intelligence (AI) technologies are currently improving rapidly and gaining popularity among both students and educators. AI has proven popular for its potential to improve the processes of teaching and learning [1]. AI is a software-based technology capable of imitating actions associated with human intelligence [2]. In addition, it blends technologies, thereby enabling machines to mimic the cognitive activities of the human brain to achieve both problem solving and learning [3]. To date, two types of AI models have been identified [2]. First, the predictive model, which can identify and classify information, and second, the generative model (GenAI) that can create and produce content, that is, ChatGPT [4]. Released in November 2022, ChatGPT is an AI language model designed by OpenAI [5] that also incorporates a chat function, produced by a Generative Pretrained Transformer using deep learning (i.e., a form of machine learning) to process and generate natural language texts [6] exploiting a considerable database comprised of human conversation [7]. ChatGPT can both produce text and interact textually, and its design realistically imitates human engagement during vocal interactions [8]. Owing to being easy to access and having a user-friendly interface that generates highly advanced language, ChatGPT has proven popular for language learning [9].

Using AI outside the classroom can enhance language learning. AI can allow English as a Foreign Language (EFL) learners to practice language at their own pace as it delivers a personalized learning experience [10,11], adapting its content to meet learners’ needs [12–14]. AI can assist learners with improving productive skills (i.e., grammar, vocabulary, speaking and pronunciation) and provide tailored automated feedback on language use [15]. Furthermore, AI can also be used to improve writing fluency [16] by enhancing efficiency as it applies to various aspects of writing, including: (1) checking grammar and syntax; (2) providing instant feedback; (3) facilitating the generation of ideas; and (4) offering personalized instructions [17]. Moreover, AI can sustain language learning engagement by increasing learners’ motivation and enjoyment [18] and offer an attractive and stress-free setting [19].

AI technologies require the user to have digital literacy, that is, practices creating meaning using digital tools [20], and a knowledge of routine literacy activities [21]. The concept of digital literacy is particularly relevant in contexts where educators, who are often described as “digital immigrants” [22], may have different levels of technological familiarity compared to their digitally native students. These are categorized as “vernacular” when they originate in everyday life, unregulated by the procedures and formal regulations applied within institutions [23]. The practices involving digital technologies can influence how individuals use and learn languages, including English [24], and can be viewed as sustainable due to their ability to maintain engagement [25].

Drawing on the concept of “vernacular literacy practices” in the paradigm of the New Literacy Studies (NLS) [21,26,27], this study investigates the vernacular literacy practices of EFL students. This paradigm focuses on specific practices, rather than decontextualized skills, so as to effectively examine what people actually do when learning English with AI. As defined by Barton and Hamilton [21], practices in this context refer to literacy activities that are voluntary, rather than mandated by formal education, self-generated, initiated by the individual user, and intrinsic to everyday life. They have originated from experiences at home and within communities of individuals. They may be dynamic, linked to the value and identities of the individual, and variable according to their needs and social purposes. In this study, the vernacular literacy practices of EFL students, refer to the informal ways in which students use AI tools to perform their everyday activities and fulfill their English language needs. Thus, the current study examines the underexplored area of informal AI-mediated English literacy practices among EFL learners. This study contributes to the NLS framework by extending the concept of vernacular literacy practices into AI-mediated contexts. Furthermore, our study highlights how AI tools function as dynamic literacy environments that shape language use, learner agency, and everyday meaning-making in informal settings. As conceptualized by Liu et al. [28], critical digital literacy extends beyond technical proficiency to interrogate how power relations, access inequalities, and learner agency shape engagement with digital technologies. In the context of AI-mediated language learning, a critical lens examines questions such as: Who benefits from AI-generated content? How do learners evaluate the authority and accuracy of AI outputs? To what extent do AI tools empower or constrain learners’ linguistic choices? We adopt this critical perspective to complement the NLS framework, thereby allowing for an analysis not only of what literacy practices emerge but also of how social and technological power dynamics influence those practices. Specifically, we address the following research question:

### RQ: What are EFL students’ vernacular literacy practices in English when using AI outside the classroom?

We also draw on critical digital literacy frameworks [28] to examine how issues of access, agency, and power shape learners’ engagement with AI-mediated literacy practices. In this context, AI-mediated English literacy practices are understood as socially situated, technology-mediated language activities that emerge through interaction with AI tools in everyday settings.

## Literature review

### Benefits of AI in education

The existing literature includes research into the uses of AI language models in education, and focuses on the perceptions of users. For instance, Adeshola and Adepoju [29] identified a number of benefits concerning the uses of ChatGPT as an educational tool, as reported in tweets: (1) effective problem solving; (2) enhancing students’ engagement and understanding; (3) generating high standard content; and (4) providing a dynamic learning opportunity. Faisel [30] concluded that employing ChatGPT in higher education (HE) can help to develop personalized learning, create content, translate between languages, and assist with language learning. Kuhail et al. [31] reported that chatbots apparently empowered learners by enhancing their understanding of challenging written work, as well as customizing learning materials and suggesting personalized learning pathways.

In addition, a number of quantitative studies have examined the benefits of AI in HE from the perspective of students. For example, Farhi et al. [7] conducted a questionnaire survey with 388 students to explore the use of ChatGPT in two universities in the United Arab Emirates. The participants considered ChatGPT a revolutionary instrument that improved their essay writing, ultimately assisting them in completing their assignments and translations. Similarly, Pavlenko and Syzenko [32] investigated students’ perceptions regarding the impact of using ChatGPT as a learning tool at two Ukrainian universities. They collected data from 247 students via an online questionnaire and found that most valued the use of ChatGPT as a learning tool. They noted that it assisted them in writing their assignments quickly and enhanced the quality of their work by correcting their language, augmenting the quality of their literature reviews, and assisting them with presentations and brainstorming. Syahrin and Akmal [33] adopted a focus-group approach to investigate students’ attitudes regarding the use of ChatGPT at a university in Oman. Students viewed ChatGPT as a helpful tool that increased their autonomy in overcoming academic challenges, boosted their confidence in their ability to handle complicated topics, and assisted their understanding of complex assignments, thereby helping them to brainstorm ideas and improve their writing. Buragohain and Chaudhary [34] explored ethical and pedagogical perspectives on ChatGPT use in HE and highlighted both opportunities for personalized learning and concerns related to academic integrity.

These studies indicate growing recognition of AI’s pedagogical value in HE, where researchers have employed qualitative and quantitative measures to collect the views of students and educators with regard to AI. They reported that AI tools can develop problem solving skills, support autonomous and personalized learning, improve writing quality, understanding of complicated materials, and enhance engagement. These findings, which were collected by analyzing social media posts, case studies, large-scale questionnaires, and focus groups indicate that AI is viewed as an essential instructional supplement with the potential to streamline academic tasks and scaffold language learning.

### AI in language education

Research into AI and language learning, particularly in relation to English, has increased [35], with growing evidence that the incorporation of various artificial language models can enhance the learning experience. Kohnke et al. [8] examined the affordances of ChatGPT in language learning and teaching, finding it convenient, and providing: (1) an avenue to practice at all times; (2) robust linguistic input; (3) human-like conversations; (3) personalized learning; (4) understanding of the meaning of vocabulary in context; (5) correction and clarification of language errors; (6) creation of input in different genres; (7) dictionary definitions; (8) opportunities to use words correctly in sentences; and (9) translation options. Jen and Salam [36] reviewed various studies relating to the use of AI in essay writing and concluded that AI tools could enhance learners’ writing skills, ultimately enabling them to generate ideas, while also evaluating their essays and offering feedback. Liu and Zhao’s [37] scoping review of AI-mediated informal language learning identified key factors such as learner motivation, engagement, and technological accessibility. Liu et al. [38] further examined AI-mediated informal digital learning of English and demonstrated that learner motivation and enjoyment significantly influence engagement with AI tools.

Several studies have employed quantitative measures to examine the use of AI in EFL. Mohamed and Alian [39] employed a questionnaire with 64 EFL students to examine the processes associated with language learning. The students expressed generally positive attitudes toward the use of chatbots. They explained that such technology provides a low-threat environment in which to practice English, subsequently enhancing their confidence and enthusiasm, and encouraging them to engage in interactive conversations. Cai et al. [40] employed a questionnaire to explore the factors influencing the attitudes of 458 students toward ChatGPT as a tool for learning English in a Chinese university. The participants considered it efficient and easy to use, and noted that it offered them a positive experience when used alongside other resources to support the learning process.

Several studies have also examined the attitudes of language learners in HE settings regarding the effects of using AI technologies to develop their writing skills in an EFL context. For example, Malik et al. [3] used an online questionnaire to explore the attitudes of 245 Indonesian students attending different tertiary institutions regarding the use of AI for writing academic essays in English. The students considered these tools improved their writing abilities and their academic integrity by: (1) supporting the writing process; (2) generating content; (3) checking their grammar; and (4) translating between languages. Similarly, Al-Zubaidi et al. [41] used a questionnaire to examine the impact of ChatGPT on the academic English writing of university students in Morocco. The participants considered ChatGPT an effective and time-saving tool, which benefited their academic English writing in areas such as: (1) developing sentence structure; (2) paraphrasing content; (3) reviewing grammar; (4) providing instant feedback on writing tasks; and (5) correcting errors.

A number of further studies have examined the influence of AI on EFL learning in HE settings, using an experimental design. For example, Alharbi [42] explored the use of Google Translate (GT) (i.e., an AI-enabled translator) to develop the writing skills of 234 students in three HE institutions in Saudi Arabia. The students’ writing drafts were assessed both with and without the use of GT using pre- and post-tests, and their perceptions were investigated. The students’ writing was improved by GT as it reduced their language errors and increased the complexity of sentence structures. The majority of the students valued the option to use GT and expressed an intention to continue using it for their English assignments, in particular as it (1) improved their capacity in English, (2) helped them overcome challenges, and (3) improved their grades. They also stated that they depended on GT for translation, despite their teachers’ instructions not to do so. In the Saudi context, Wahdan and Buragoahian [43] examined writing pedagogy in EFL settings, further emphasizing the need for structured approaches to support student writing development.

In terms of AI’s effect on grammar knowledge, using mixed methods of pretest and post-test, as well as a focus group, Kucuk [44] examined the benefits of using ChatGPT to learn grammar among university students in Iraq. The students were divided into experimental and control groups. Students in the control group studied grammar using traditional methods, while the experimental group used the interactive interface of ChatGPT for a seven week period. Students in the latter group accessed ChatGPT whenever they encountered any difficulties with English grammar or spelling, and received instant answers tailored to their specific needs, including corrections of their grammatical errors. The experimental group outperformed the control group in the post-test. Furthermore, the students reported that ChatGPT was easy to access and enhanced their comprehension of complex grammatical structures, while simultaneously increasing their motivation to learn grammar.

In terms of AI’s effect on the speaking skills, Zou et al. [45] explored the use of AI speaking apps to practice speaking in English with 70 Chinese university students (experimental and control groups). The experimental group engaged with an interactive AI speaking app over a period of five weeks. Engaging with AI speaking apps with interaction significantly enhanced the proficiency of participants, improving their spoken fluency, pronunciation, grammatical accuracy, and presentation skills. Zou, Lyu, et al. [46] conducted a further study to understand the views of university EFL students regarding the use of AI apps to enhance their speaking ability when focusing on “English for Academic Purposes (EAP) Talk.” The study implemented mixed methods comprising of questionnaires and follow-up interviews. The researchers also found the participants regarded EAP Talk as beneficial, as it was easy and enjoyable to use. Furthermore, the participants also felt AI evaluated their oral production accurately, was useful for correcting their errors, motivated them to improve their pronunciation and learn more vocabulary, and increased their opportunities to practice speaking without fear of negative peer evaluation.

These studies reveal that AI can be used as an effective and multifaceted resource in the area of English language learning to develop students’ outcomes and enhance their skills. The quantitative design revealed positive attitudes toward AI technologies, with learners reporting enhanced writing abilities and greater confidence, while experimental studies demonstrated that the use of AI-supported-instructions improved proficiency in multiple skill areas. As Soyoof et al. [47] argue, AI should not only be viewed as a tool, but also as a discourse that shapes how literacy practices are constructed and experienced. However, an exploration of the use of ChatGPT from the perspective of students [7], including those in HE [30], employing qualitative [3] as well as quantitative and mixed methods [9] is crucial. The current study investigates the underexplored area of vernacular, AI-mediated English literacy practices among EFL learners focusing on informal literacies in English.

## Methodology

### Participants

The participants were 12 postgraduate students attending a single university in Riyadh, Saudi Arabia. The data collection started on December 5^th^, 2024 and lasted for five successive weeks. The data collection procedures, including the duration, participants, and tools used, are summarized in Table 1. As male and female students are separated in most educational settings in Saudi Arabia, only female students were invited to participate. The researcher was female and inviting male students would not yield insightful data, as building rapport would be a challenge for cultural and religious reasons. These female students were accessible to the researcher and had previously reported using AI in their daily lives. The sampling strategy was purposive [48] and the sampling criteria were as follows: (1) postgraduate students majoring either in Human Resources Management (HRM) or Internal Audit and Governance, and (2) who had experienced using AI apps or websites in English over a period of at least two months (Table 2). The combination of purposive, chain, and convenience sampling was employed to address the research aim effectively. Purposive sampling was first used to select participants who met the abovementioned specific criteria. This ensured that all participants had relevant experience with AI-mediated English literacy practices. Chain sampling (snowball sampling) was then used, where initial participants referred other eligible students from their networks, thereby helping to reach participants who were not easily identifiable through other means. Finally, convenience sampling was applied within university premises to recruit additional participants who were readily available and willing to participate. This multi-strategy approach is common in qualitative case studies where access to specific populations is limited, and allowed the researcher to achieve the target sample size of 12 participants within the available timeframe.

**Table 1:** Summary of data collection procedures.

Data collection tool	Participants ID	Tool date	Tool length (hr:min)	Number of Screen recording videos
Focus group session (1)	P1, P2, P3, P4, P5, P6	5^th^ December 2024	01:10	X
Focus group session (2)	P7, P8, P9, P10, P11, P12	13^th^ December 2024	01:30	X
Retrospective interview 1	P1	8^th^ January 2025	00:20	1
Retrospective interview 2	P2	19^th^ December 2024	00:22	1
Retrospective interview 3	P3	9^th^ January 2025	00:23	1
Retrospective interview 4	P4	3^rd^ January 2025	00:19	1
Retrospective interview 5	P5	30^th^ December 2024	00:21	1
Retrospective interview 6	P6	22^nd^ December 2024	00:24	1
Retrospective interview 7	P7	15^th^ December 2024	00:25	1
Retrospective interview 8	P8	28^th^ December 2024	00:21	1
Retrospective interview 9	P9	7^th^ January 2025	00:16	1

**Table 2:** Participants’ basic demographic information.

Participants ID	Age	Major	Experience with AI (in months)
P1	31	HRM	3
P2	26	HRM	5
P3	32	HRM	3
P4	27	HRM	4
P5	27	Internal Audit and Governance	5
P6	25	Internal Audit and Governance	8
P7	24	HRM	6
P8	29	HRM	5
P9	26	Internal Audit and Governance	8
P10	30	Internal Audit and Governance	3
P11	31	Internal Audit and Governance	4
P12	25	HRM	7

Approval was obtained from the Research Ethics Review Committee at King Saud University (IRB: KSU-HE-24–1045) on November 26, 2024. The participants were recruited within university premises. The researcher explained the research objective to the participants, including what it entailed, and also distributed a consent form explaining that all participation was voluntary, and withdrawal from the research was possible at any time, while also confirming that the data would remain confidential and anonymous. Respondents gave written consent for review before starting the focus groups and retrospective interviews.

The two majors (HRM and Internal Audit and Governance) were selected because both programs require substantial English reading and writing for academic assignments, and students in these disciplines frequently engage with AI tools for academic and workplace-related tasks. The use of a female-only sample reflects the gender-segregated educational context in Saudi Arabia, where males and females study separately. While the sample size of 12 participants is relatively small, qualitative research suggests that data saturation in homogeneous samples can be achieved with 9–12 participants [49,50].

### Design: qualitative case study

This study applied a qualitative research design as this can provide insights into the social and situational factors that shape aspects of language use [51]. This proves valuable when describing participants’ behaviors, perceptions, and experiences [52]. In addition, a case study format was used, as this is considered appropriate for developing an in-depth understanding of the features of a particular community [53,54], such as the vernacular literacy practices that occur in online platforms [26]. The data collection methods consisted of a focus group and retrospective interviews. To create a spontaneous conversational discussion in which each participant fully understood the purpose of the research tools and any relevant concepts, the researcher first informed the participants in detail about the research aim, along with the interview procedure and related terminology pertaining to AI. The focus group and interviews were conducted in Arabic to ensure fluent and accurate communication [55]. Moreover, the participants were postgraduate students, with limited time, who had agreed to participate in an online study only. Thus, Zoom meetings were considered suitable for collecting the qualitative data required [56].

### Focus group

The focus group was employed as the first research method to encourage participants to generate ideas together, so as to challenge and inspire one another to react to the questions proposed by the researcher [51]. This technique is generally used to elicit differing viewpoints about a particular issue, as group interactions tend to encourage the participants to express their views more effectively than individual interviews [57].

A total of two sessions of focus groups were held, with six participants in each. The duration of the first and second session was 70 and 90 minutes, respectively. The researcher used open-ended questions, which were initially piloted with a group of six participants to ensure clarity and the smooth flow of the focus group interaction. These were then edited, and modified questions added (see Appendix for final questions). The researcher also invited the participants to share screenshots to illustrate their responses.

### Retrospective interview

The second research method consisted of retrospective interviews, in which the participants were requested to verbalize their thoughts while performing a task ([51]. Only nine of the 12 students who had participated in the focus group sessions agreed to participate, as they were busy balancing their social, professional, and study commitments. They were invited to record their screens to provide examples of how they were using AI apps or websites in English. They were then asked the following questions: (1) How do you access the app or website? Are there specific steps? Please describe what you do in detail. (2) What did you do after that? Describe the steps you followed. (3) Why do you use this app or website? Each participant presented one screen recording of her activity.

### Data analysis

The audio recording of the focus groups, and the retrospective interviews were transcribed for the purpose of analysis. The focus group and retrospective interviews were conducted in Arabic and the participants sometimes used English to express their experiences, although they always used English when screen recording their AI activity. The data were not translated into English in the analysis phase as the raw data was expected capture their essence and nature. The excerpts were translated by the researcher (who is fluent in both English and Arabic) when presenting the results. To ensure translation accuracy, selected excerpts were reviewed by a bilingual expert fluent in both Arabic and English. To ensure the credibility and accuracy of the findings, member checking was conducted with selected participants. The texts, screen recording videos, and screenshots were all uploaded to ATLAS.ti software to assist with the analysis, and content analysis was used to condense the data by combining inductive and deductive codes [58]. Content analysis was chosen to facilitate systematic identification of vernacular literacy practices across multimodal data. The choice of a deductive framework was guided by the research question and the concept of vernacular literacy practices, where each activity on AI in English performed by the participants was coded. Inductive codes were also used and emerged from the data denoting AI-mediated activities in English. Codes relevant to the research question or previous literature were attached to segments in the transcribed text, and the screen recording videos were connected to the retrospective interview transcription data. The segments were quoted first, and then codes were attached to the quotations on ATLAS.ti (version 25). In addition, to help manage the data for retrieval purposes and assist with data analysis, the screen recordings provided by each participant were linked to the transcript of the corresponding interview.

The data analysis procedure involved the following steps: (a) transcribing the audio data in the text in MS word files; (b) uploading the files and screen recording videos to ATLAS.ti.; (c) reading the text several times, and watching the screen recording videos repeatedly to develop a general impression of the data; (d) identifying segments related to the concept of vernacular literacy practices and the research question, and labeling these with codes; (e) interpreting and sorting the codes by merging similar codes, renaming the codes, assigning subcodes and group codes in ATLAS.ti.; (f) creating themes from the group codes to answer the research question, as a theme is “an extended phrase or sentence that identifies what a unit of data is about and /or what it means” [59] (see Table 3). An example of how raw data were transformed into codes, categories, and themes is presented in Table 3.

**Table 3:** Excerpt of the content analysis used to analyze the screen recording transcript of the example provided by Participant 4.

Meaning Unit	Condensed Meaning Unit [sub -code on ATLAS.ti]	Code	Category [code group on ATLAS.ti]	Main Category	Theme
P4: I want to ask you something. I have an interview tomorrow and I want to know how to prepare myself for the interview. Do you have any advice for me? ChatGPT: absolutely first things first. It’s totally normal to feel nervous before an interview, to start try to learn as much as you can about the company you’re interviewing with. Check out their website and any recent news about them. That way, you can show you’re really interested. Also, think about some of the skills and experience they are looking for in the job description. Can you think of specific examples from your past where you used the same skills to talk about? P4: but I don’t know! I don’t have any prior experience; how can I show them that I’m ready to work?	Case: Engage in a verbal dialogue to practice for a job interview	Practice job interviews with ChatGPT	Provides advice for job interview Ask for advice ChatGPT: Smartphone	Vocational life	Practices to serve daily life

To ensure inter-rater reliability, the researcher invited two colleagues to analyze and code the focus-group data and three retrospective interviews using the established deductive framework. Inter-coder reliability was established as the independent coders discussed similarities and discrepancies in the coding, refined the coding scheme, and reapplied the codes. For example, the two independent coders used the categories of “work-life” and “vocational-life” to label the codes “assist in work duties” and “provide advice on job interviews,” whereas the researcher had used the category “personal life.” The coders discussed the categories and explained that these were distinct from “personal life” and suggested using the category “vocational life” as an alternative. The subcodes of the code “assist in work duties” were “assist in resumes” and “write emails.” The researcher was then convinced and agreed to assign the categories for the codes and subcodes as attached to the data segments. The categories of “personal life” and “vocational life” were then assigned a theme of “practices to serve daily life” (see Fig 2). The final coding schemes were updated, and the two coders and the researcher matched 85% in their coding scheme, thus reflecting a high level of validity. The 85% agreement was calculated based on 45 coded segments across four transcripts (two focus group and two retrospective interview transcripts). This percentage exceeds the commonly accepted threshold of 80% for inter-coder reliability in qualitative content analysis [58,59]. The final step was to create a tree diagram for each theme from the network feature on ATLAS.ti to assist with presenting the results. The tree diagram is presented in the following section. The researcher acknowledges that the absence of independent back-translation may introduce interpretive bias. However, multiple reviews of translated excerpts and participant validation through member checking were used to enhance credibility. The translation process involved (1) researcher translation, (2) bilingual expert verification of selected excerpts, and (3) participant member checking of translated quotes.

## Results and discussion

This section discusses the results in relation to the research question and previous literature.

### RQ: What are EFL students’ vernacular literacy practices in English when using AI outside the classroom?

The vernacular literacy practices in which the participants engaged when using AI in English are categorized according to their usage purposes in three domains: (1) practices for English language development; (2) practices to serve daily life; and (3) practices to serve academic life. Approximately 50% of AI engagement in the English language was undertaken to improve their capacity in English, and many were interrelated and intertwined.

Regardless of their usage, this study identified a number of common characteristics of these vernacular AI-mediated literacy practices. The literacies were convenient, as all the participants reported that accessing AI is easy from any device connected to the Internet. They access AI from their smartphones, either in the form of apps or website. They explained this method saved time, as their smartphones were always with them. This finding aligns with previous research, which also determined AI to be time-efficient [39–41]. Another characteristic was informal learning methods, with participants stating:

“I learned it from X. I mean Twitter. No one taught me to use it.” (P11)

“I saw people talking about ChatGPT on TikTok and I used it after that.” (P1)

“I knew about ChatOn AI from TikTok.” (P12)

This suggests these vernacular AI-mediated practices were embedded in the everyday lives of these EFL students, as they were motivated to learn with AI informally; and learned about using AI accidentally from social media, mainly TikTok and Twitter. Their literacies were self-generated, autonomous, and not shaped by the educational institutions in which they were studying. This also suggests these practices are embedded in their social and cultural lives as the participants engaged with them to perform activities in response to their personal interest.

Another participant noted that these literacies were frequently associated with real-time interactions, for example: “I use the app because it gives fast and instant answers. I can ask and interact with it on the spot” (P9), and “The best part about it is the dynamic interaction. I can type, use the mic, or simply listen after writing my question” (P3). This suggests these literacies were multimodal and dynamic, involving text and audio. AI-mediated literacies enabled users to type the text, listen to the audio, and speak using a microphone. This can be linked to the dynamic, multimodal vernacular literacy practices that constantly evolve and change in different social settings [26]. These findings are in line with the characteristics of vernacular literacy practices [21,23] as they were not regulated by universities and were acquired in an informal manner. This finding extends NLS by demonstrating that vernacular literacy practices in AI-mediated environments retain core characteristics of traditional vernacular literacies (voluntary, self-generated, informal) while introducing new affordances such as real-time multimodality and personalized interaction.

The concept of vernacular literacy practices, when mediated by AI in the English language, can then be expanded to include all the activities in the English language that occur on AI apps or websites, as performed by the EFL students. Such practices refer to the activities of typing, copying, pasting, reading, speaking on the microphone, listening to AI-generated audio, or engaging in a lengthy dialogue on AI. These include the use of touch screens on portable devices – iPad or smartphones – which are connected to the Internet to type the queries, select, copy and paste texts from AI apps to other apps/programs and vice versa. These literacies were multimodal, synchronously dynamic, self-generated, involved learning informally, and were not regulated by educational institutions as they were carried out for specific purposes.

### Practices for english language development

**Fig 1:**
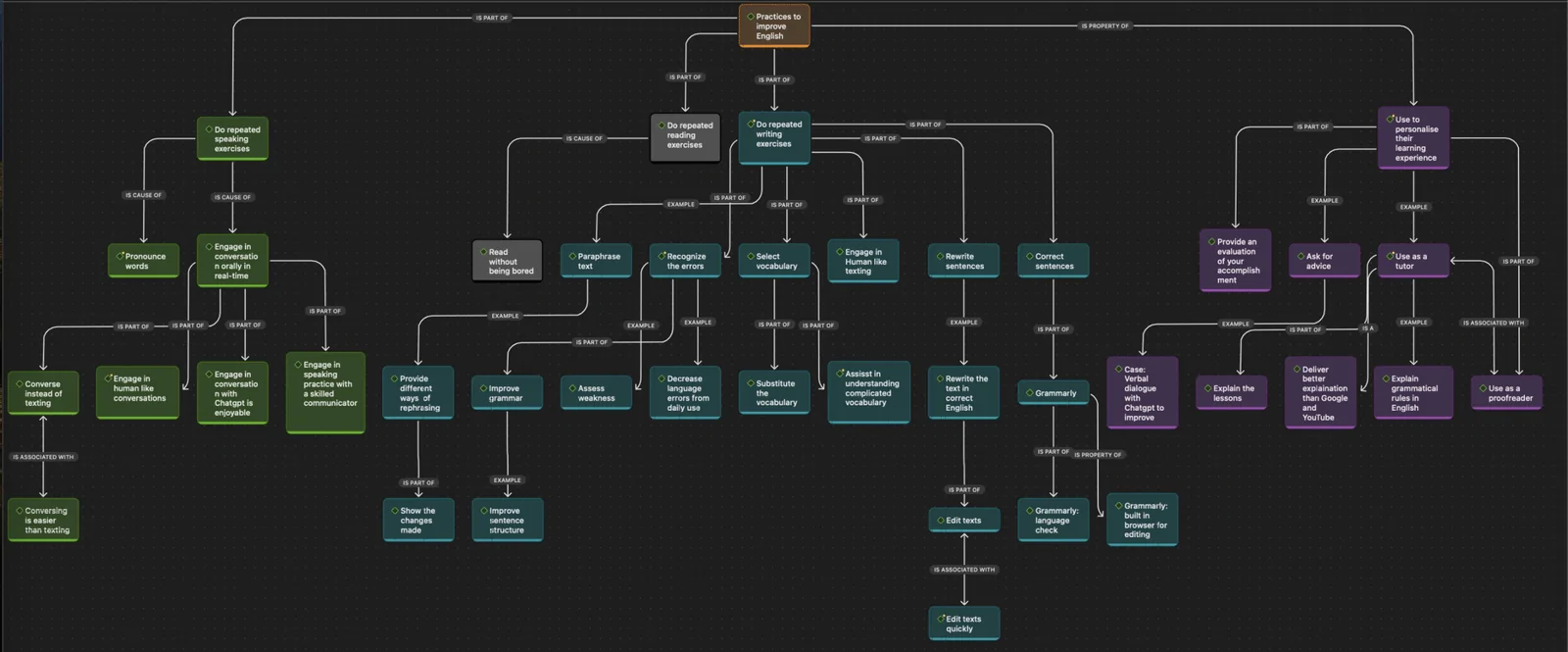
Network feature on ATLAS.ti: Codes and subcodes related to the theme “practices for English Language Development”.

The data indicated that the postgraduate students engaged with AI to practice their English proficiency, including: (a) repeated speaking exercises; (b) reading exercises; (c) repeated writing exercises; and (d) using AI to personalize their learning experience (see Fig 1).

As indicated above, one of the practices noted by the postgraduate students involved repeated speaking exercises to improve their English. The participants engaged in oral conversation in real-time with AI, as well as using it to improve their pronunciation. They mentioned conversing was easier than texting, and that they enjoyed engaging with ChatGPT:

“…talking with ChatGPT is much easier than texting. It is faster and I don’t need to look for the letters from the keypad.” (P1)

“…I talked to ChatGPT out loud for the first time this morning, and honestly, it was fun! It was much easier than writing or texting, and I liked that I didn’t have to type in the chat box during our conversation, which saved me time.” (P8)

The instantaneous replies provided by AI can provide rich dynamic learning [29], which suggests the conversational abilities of postgraduates may support speaking development. This is because the participants believed it resembled conversing with a human being fluent in English. For example,

“…You know, with the ChatGPT Plus subscription, the voice is actually human instead of sounding like a robot. It’s way different from the regular voice, which feels more robotic. The human voice is really natural and speaks smoothly.” (P1)

“It’s the only place to practice speaking English with someone who is really fluent.” (P10).

This finding is consistent with previous studies, further indicating the potential of AI to support speaking proficiency levels and pronunciation, as well as increasing the chances of practicing speaking skills [19,45,46,60]. This real-time synchronous practice, where students engage in human-like conversation, can potentially offer fluency gains, but can also minimize spontaneous interactions in real-time with English speakers. Moreover, language students may improve in terms of their automated responses but may not necessarily improve their adaptive conversational abilities.

In addition, the participants noted that they felt a greater willingness to undertake reading exercises repeatedly. They explained that AI provided them with a platform from which to practice their reading skills while maintaining interest, for example:

“It helped me read in English without noticing how much time was passing or even realizing I was reading. Usually, I get bored quickly with books, but now I stay interested and don’t feel the time fly by.” (P11)

“I use it to practice my English reading skills. Although I’m already advanced and don’t need to study it formally, I want to keep practicing ensuring I don’t lose my proficiency.” (P5)

The increased engagement reported by students could be due to AI convenience. The reading practice without guided tasks may not target critical reading skills or comprehension strategies. However, this suggests that AI has the potential to motivate students only when their engagement declines, which resonates with Borkovska et al. [61] and Mohamed and Alian [39], who found that AI technologies can enhance motivation and enthusiasm.

As well as engaging in repeated speaking and reading exercises, the participants reported that AI, in the form of Grammarly, helped them to correct their sentences in written English. Participant 7 said: “I’ve turned on Grammarly in my browser so I can check my English every day.” This finding resonates with Alharbi [62] and Borkovska et al. [61], who suggested Grammarly can serve as a vital technology to assist with language learning. AI enabled the participants to select the vocabulary by substituting words, and assisted them to understand complex vocabulary, and identify their errors. Participant 9 provided a screenshot to illustrate how AI explains changes it makes to texts.

Participant 9 stated, “I’ve seen the mistakes I used to make when I wrote. Since I practice every day, I can say my writing is better now, and I make fewer errors.” This suggests that AI has the potential to provide suggestions for sentence structure and grammar due to the provision of immediate feedback. This is supported Al-Zubaidi et al. [41], Aljuaid [17], and Kucuk [44], who identified AI as an effective tool for reviewing grammar as it provides instant error correction and feedback.

The participants reported that AI enabled them to engage in human-like texting, rapidly editing their sentences into correct English. This finding concurs with earlier findings that ChatGPT simulates human-like conversation capable of enhancing language learning [8,63]. In addition, the participants used AI to paraphrase texts, employing differing styles of writing, which supports previous studies highlighting the positive impact of AI on learners’ writing skills [3,33,37]. The participants also used AI to personalize their learning experience, using it as both a proofreader and a tutor to explain the lessons and grammatical rules more effectively than Google and YouTube:

“…imagine having a language assistant who helps you improve your English for free and is available 24/7.” (P1)

“…it is a cheaper option than language classes.” (P4)

“…I used to pay for a proofreader and hire private tutors to explain English lessons to me. Now, I get answers in seconds. I am subscribed to one of the AI apps that helps with this.” (P10)

“… who needs a tutor now! Subscribing for a month is cheaper than having an hour of private teaching.” (P5)

Some participants asked for advice from AI on how to improve their English. For example, Participant 1 screen recorded engaging in verbal dialogue with ChatGPT by pressing the microphone on the bottom right of the screen and stated “I was talking with a robot. I asked what I wanted and received a reply. ChatGPT provided tips that were specially made for me.” This suggests that AI has the potential to customize language learning and tailor instructions to individual learners, which is consistent with previous studies concluding that AI offers a personalized learning environment [10,17,30,31,44]. From an NLS perspective, these English language development practices represent a new form of vernacular literacy where AI tools function as both tutor and conversation partner. Unlike traditional vernacular literacies that primarily involved reading and writing printed texts [21], AI-mediated practices incorporate speaking, listening, and real-time dialogue. This expansion of vernacular literacy to include oral-aural modalities with non-human agents extends the NLS framework to account for human-AI interaction viewed through a critical digital literacy lens; however, these practices also reveal an uneven distribution of evaluative agency: participants largely treated AI-generated corrections, translations, and feedback as authoritative, with few describing efforts to verify or question the accuracy of AI outputs. This pattern suggests that platform authority can substitute for learners’ own critical judgment, even as it supports language development.

### Practices to serve daily life

**Fig 2:**
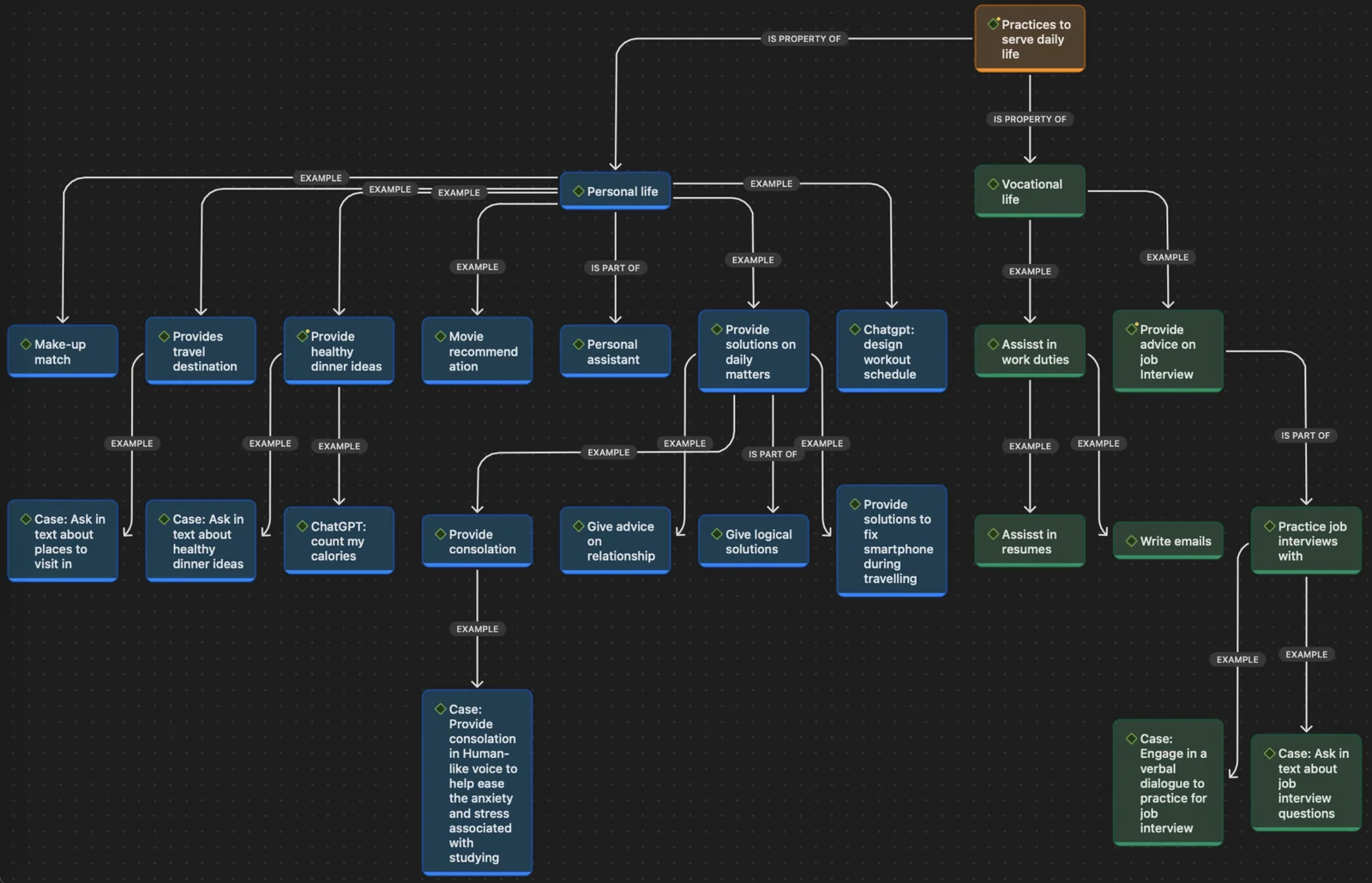
Network feature on ATLAS.ti: Codes and subcodes related to the theme “practices to serve daily life”.

The data indicated that the postgraduate students engaged in AI practices to benefit their daily lives in both personal and vocational areas (see Fig 2).

The participants also reported that AI assisted them with their work, including writing emails and resumes. Participant 8 stated: “…ChatGPT helped me out when my manager nominated me for a higher position. I needed a strong resume, and it assisted me in creating one that looked professional!” In addition, most reported using AI to create emails to communicate in their work:

“…I use ChatGPT every day, just like my friends, to help with writing emails. I usually write part of the email myself and then ask ChatGPT to finish it and change the style. I use this tool as an employee, not just as a postgraduate student, since I’m balancing both roles at the same time.” (P12)

“… I am an employee too, and when I’m busy, I ask ChatGPT to help me edit my emails. I just copy, paste, and send them without reading them.” (P9)

These accounts demonstrate the benefits of using AI to write emails and resumes, but also provide evidence that participants are relying heavily on AI. They gave AI the responsibility of creating a professional resume as well as evaluating the format of an email. Although this provides short-term efficiency by saving time, their opportunities to develop long-term literacy in drafting, reviewing, and editing their own writing are reduced. Participants often send AI-generated emails without reading them. However, they did not express concerns about information quality, and this remains an area for future inquiry.

The participants asked AI for advice about job interviews and also when practicing for interviews. Participant 4 screen recorded how she practiced engaging in a verbal dialogue with ChatGPT to prepare for a job interview and obtain useful tips. In addition, Participant 5 screen recorded her textual engagement with ChatGPT to prepare for a job interview, and noted that she first asked about the interview questions and their answers, and then practiced answers and memorized them to sound more natural. While memorizing AI-generated responses may support short-term performance, such approach does not promote deeper communicative competence or authentic language use and may limit meaningful engagement with the language.

Moreover, the participants drew on practices to enhance their personal lives, including matching makeup shades from different brands, and requesting assistance when traveling abroad. Participant 8 screen recorded her textual interaction with ChatGPT in which she asked about flights to Spain and places to visit. She also mentioned that ChatGPT provided her with a link to a website to book her airline tickets.

AI also assisted the participants with improving their lifestyles, offering ideas for healthy dinner recipes, counting their calories, and designing workout schedules. Participants also reported that AI acted as a personal assistant, recommending movies and providing solutions on daily concerns, including offering relationship advice and reassurance:

“I had an issue with a friend, so I decided to ask ChatGPT how to fix it, and it actually gave me a great solution. Then, while I was traveling, my phone stopped working. I borrowed my sister’s phone to reach out to ChatGPT again, and it really helped me get my phone working again.” (P3)

“…I was feeling quite overwhelmed by my studies and really needed someone to support me and offer encouragement. So, I reached out to ChatGPT. It spoke to me in a human-like voice, and reassured me that the struggles I’m facing right now will eventually transform into happiness once I have completed my degree and secure a successful job in the future.” (P1)

The excerpts demonstrate that the participants sought the assistance of AI even for issues of a personal nature, such as with emotional reassurance and consolation. AI can help with everyday tasks. This may tentatively suggest a potential risk of overreliance on AI for psychological support, although the present study did not measure psychological outcomes directly. Further research is therefore needed to determine whether such practices replace or complement human support. The participants treated AI as a human being and the expression “spoke to me in a human-like voice” highlights such view. These AI-mediated literacies permeated the physical, emotional, and online life of these participants.

The vocational and personal practices observed in this study illustrate how AI-mediated vernacular literacies blur traditional boundaries between public and private, work, and personal life. While Barton and Hamilton [21] conceptualized vernacular literacies as embedded in local communities, these findings suggest that AI tools enable vernacular practices that transcend physical communities, thereby creating new “algorithmic communities” where literacy practices are shaped by AI responses rather than solely by human interaction. These patterns of reliance also illustrate a critical digital literacy dimension of dependency: participants rarely reported verifying AI-generated content in personal or vocational contexts, ultimately extending trust to the platform in ways that may narrow opportunities for independent judgment and gradually reshape learner agency.

### Practices to serve academic life

**Fig 3:**
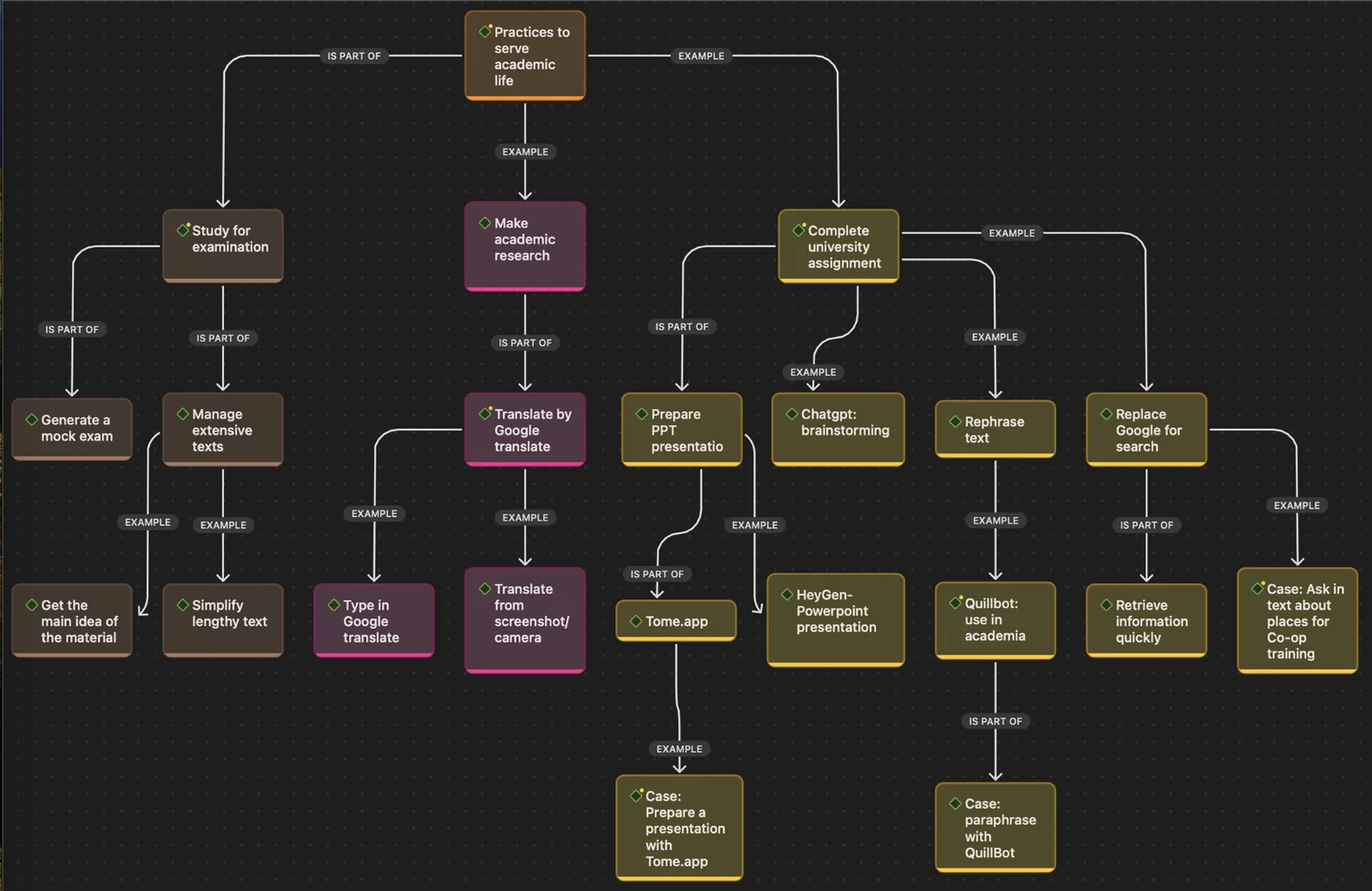
Network feature on ATLAS.ti: Codes and subcodes related to the theme “practices to serve academic life”.

The data indicated that the participants used AI in English to assist function in their academic lives (see Fig 3), including: (1) studying for examinations, (2) undertaking research, and (3) completing university assignments.

The participants also reported that using AI assisted them when studying for their examinations by generating mock tests, and also managing extensive texts, either by uploading content to AI in the form of a PDF file or writing the name of the grammatical rule and using particular prompts. For example:

“… I had an exam coming up, and I wanted to prepare with a mock exam. So, I uploaded the material in PDF format and asked AI to create multiple-choice questions along with their answers.” (P7)

“… I uploaded the slides about the present simple tense, and I wrote in the box ‘make multiple-choice questions’. Actually, it helped me create questions that were easy, medium, and hard.” (P2)

In addition, the participants explained that uploading lengthy texts to AI helped them to simplify the information contained therein, and to understand the main ideas contained in the material.

Furthermore, they used AI to assist with academic research, in particular translating from English to Arabic. Participant 6 reported that she used GT to translate excerpts of research from English into Arabic. She screen recorded her activity by opening the browser, typing her topic of interest into the Google Scholar research box, then clicking on one of the links leading to the research she wished to examine in detail. She then screenshot the text and went to GT to translate the excerpt: “… It was a long process, but it really helped me with my research paper” (P6). This aligns with previous findings that AI technologies assist with translation [3,7,30,42,64].

The participants further reported using AI to assist them when completing their university assignments, including designing presentations in the English language and brainstorming ideas. Participant 3 mentioned Tome as a user-friendly website that offers templates to design a presentation and includes the option to edit them. Participant 4 reported that Tome prepared a complete presentation for one of her university courses:

“...It created a polished and visually engaging presentation, and the best part is that you don’t need to be a graphic designer to do it. I no longer stress about making slides or using PowerPoint; I can get everything done in just seconds.” (P4)

Participant 8 mentioned that HeyGen assisted her in creating a PowerPoint presentation with its ability to provide textual content supported by images generated by AI. This finding corroborates those from previous studies, further indicating that AI can both generate ideas [17,33,36] and enhance presentations [32].

The participants also mentioned using AI for paraphrasing texts, with Participant 7 screen recording her activity when using a QuillBot AI-powered website to paraphrase a text. She mentioned that it allowed different modes of paraphrasing and provided the changes made to the text in different colors. The site also suggested additional suggestions for rephrasing when the user clicked on the sentence. Participant 7 stated “…I really wish I had known about this while I was pursuing my bachelor’s degree. It rephrases the text in different styles according to the mode you choose and presents various vocabulary options from which to select.”

The participants also noted that they use AI to search in English to assist them with their university assignments. Participant 2 mentioned that she used ChatGPT to search for the best companies for co-op training during her academic studies. She was required to train for a specific period of time and screen recorded her textual engagement with ChatGPT. She stated, “…I’ve shifted to using ChatGPT for my information searches rather than Google. I find that it retrieves information quickly and allows for interactive discussions, which helps me gain additional explanation on the subjects I’m exploring.” (P2). Although the Google search engine is partially powered by AI technologies, most of the participants did not consider it an AI app, in the same way as ChatGPT and ChatOn AI. This suggests that they expressed a preference for searching with AI apps over the Google search engine. AI apps offer greater utility because they interact dynamically with prompts from the user and can reply in a single post, rather than directing users to existing content.

Reports of replacing using the Google search engine with AI alternatives reveals that the literacies related to the information search behavior of EFL students have evolved. Participants may have favored ChatGPT as it provided ready-synthesized output that they could read and then pose/type follow-up questions. This changed how they evaluated the search results after writing queries on Google, as they shifted from navigating to other hyperlinked sources provided by Google to ready-synthesized integrated output on AI. As the participants did not consult original sources, this may present potential challenges to information quality and critical thinking abilities. However, these observations warrant further investigation. This shift from hyperlink-based navigation to AI-generated synthesis has implications for information literacy, as learners may need new competencies to evaluate AI-generated content against original sources. This pattern reflects a critical digital literacy concern regarding platform authority and trust: as AI-generated syntheses displaced direct engagement with primary sources, participants’ capacity to independently evaluate the accuracy and provenance of information was reduced correspondingly, thus illustrating how convenience can come at the cost of learner agency. Theoretically, this study contributes to NLS by demonstrating three shifts in vernacular literacy practices when mediated by AI. First, a shift from asynchronous to synchronous real-time interaction. Second, a shift from text-based to multimodal (text, voice, audio) literacy activities. Third, a shift from community-bound to algorithmically mediated literacy practices. These shifts redefine what counts as vernacular literacy in the age of generative AI.

## Conclusion

This study explored the vernacular, AI-mediated English literacy practices among EFL students. These vernacular literacies were synchronously dynamic, self-generated, originated in response to each student’s needs and interests, and were not supervised by formal institutions. Using content analysis, such practices were classified according to practices used for English language development, to serve needs in daily life, and to benefit academic life. Those relating to improving daily life could potentially increase learners’ motivation to use English beyond educational contexts, and this would lead to better engagement and sustain their English learning, particularly as it can be undertaken on an easily accessible technology, that is, cellphones [65]. The data revealed that approximately 50% of the total AI-mediated English literacy activities were undertaken to practice English proficiency skills, and many described customized learning opportunities.

Understanding the literacies of these EFL students poses pedagogical implications for the process of English learning, since learners performed more than half of the literacies for English language development. These implications are offered as tentative, context-specific observations derived from a small qualitative case study, rather than as generalizable claims about the effectiveness of AI-mediated learning. Instructors can encourage learners to engage in AI-mediated English literacies outside the classroom to support their language proficiency and generate personalized learning content. They can also encourage them to engage in verbal interactions with ChatGPT to practice their speaking skills, as it interacts in a human-like conversational style. However, they should practice with other human beings and not rely entirely on AI-generated interaction. Instructors could also encourage learners to use AI as a private tutor because it is available 24/7, and is fast, convenient, and cheaper than private tutoring. Moreover, AI-mediated literacies can enable learners to receive immediate feedback on their writing or speaking tasks, as well as scaffolding vocabulary learning. EFL learners can use the affordances of AI to enhance the literacies related to their vocational life when writing resumes and emails without encouraging overreliance. Moreover, these practices could inform curriculum designers as they design learning materials to align with the students’ daily needs and academic needs in real contexts.

Theoretically, this study contributes to NLS by extending the concept of vernacular literacy practices to AI-mediated environments. While traditional vernacular literacies have been examined in contexts such as social media and web platforms [26,27], the present study demonstrates that AI tools like ChatGPT, Grammarly, and QuillBot function as dynamic literacy environments where vernacular practices emerge through real-time, multimodal, and personalized interactions. This extension of the NLS framework highlights how learner agency, self-generated activities, and informal learning are reshaped when mediated by AI technologies. The concept of “AI-mediated English literacy practices” thus offers a new analytical lens for understanding language learning in informal digital contexts. Complementing this, the critical digital literacy analysis indicates that these AI-mediated vernacular practices are also shaped by unequal evaluative agency: across all three practice domains, participants generally extended a high degree of trust to AI outputs, rarely interrogating the accuracy, provenance, or platform authority underlying the content they received. This suggests that AI-mediated literacy development can coexist with a narrowing of learners’ critical evaluative agency, and that fostering critical digital literacy alongside language proficiency should be a priority for both research and pedagogy.

This study has a number of limitations. First, for cultural and religious reasons, the participants were recruited by means of chains and convenience sampling of female students only. Second, the sample size was relatively small, consisting of 12 students studying in two majors at a particular university. Third, the data were obtained from focus groups and retrospective interviews. Despite these limitations, the findings are still capable of enriching our understanding of the AI-mediated vernacular English literacy practices among EFL students in Saudi Arabia.

Future studies could also include more diverse sampling by examining the behaviors of male and female EFL learners studying different majors at various universities. Other studies could also employ ethnographic tools and ask participants to document their practices in journals, to identify any shifts in the values and identities of EFL students when engaging these AI literacies. When these areas are explored and evaluated, future research could yield a comprehensive understanding of the informal AI-mediated English literacy practices within EFL contexts. The findings of the present study have important implications for multiple stakeholders. For researchers, future studies should expand the sample across diverse contexts and explore longitudinal impacts of AI-mediated literacy practices. For educators, findings from this exploratory case study tentatively suggest that integrating AI tools into language learning strategies may support student engagement and personalized learning, although this claim should be interpreted cautiously and tested with larger, more diverse samples before it can be generalized. For learners, the use of AI as a supplementary tool can support independent learning, although this should be balanced with human interaction to ensure holistic language development.

## Supporting information

S1 Appendix Focus group questions (uploaded separately). (DOCX)
