# Supplementary material for: Vernacular english literacy practices among language students using AI: A case study in Saudi Arabia
Source: PLoS One. 2026 Sep 15;21(9):e0358237. doi: 10.1371/journal.pone.0358237 (PMC13577362; doi:10.1371/journal.pone.0358237)
Supplement: Supplementary file 1 [file pone.0358237.s001.docx]

**Focus group questions**

1) What tools or technologies related to AI are you currently using in English (e.g., types of AI, usage frequency, and the purposes of using them, etc.).
2) Have you ever used ChatGPT in English in your current studies or projects?
3) Have you observed any effects of using AI on your English language skills, such as speaking, listening, reading, or writing?
4) In your opinion, does the use of AI in English have an impact (e.g., positive or negative) on your academic progress? Please elaborate on your thoughts.
5) What advantages do you perceive in using AI for English language learning? Provide a detailed description.
6) What are the disadvantages associated with using AI in English? Please explain in detail.
7) What do you consider to be the most effective way to use AI in learning English?
8) How do you think AI will be used for learning English in the future?
9) Finally, is there anything else you would like to share that has not been mentioned in the previous questions?
